# Enhanced Bilosomal Properties Resulted in Optimum Pharmacological Effects by Increased Acidification Pathways

**DOI:** 10.3390/pharmaceutics13081184

**Published:** 2021-07-31

**Authors:** Armin Mooranian, Thomas Foster, Corina M. Ionescu, Daniel Walker, Melissa Jones, Susbin Raj Wagle, Bozica Kovacevic, Jacqueline Chester, Edan Johnston, Elaine Wong, Marcus D. Atlas, Momir Mikov, Hani Al-Salami

**Affiliations:** 1 The Biotechnology and Drug Development Research Laboratory, Curtin Medical School & Curtin Health Innovation Research Institute, Curtin University, Perth, WA 6102, Australia; a.mooranian@curtin.edu.au (A.M.); thomas.p.foster@student.curtin.edu.au (T.F.); c.ionescu@postgrad.curtin.edu.au (C.M.I.); danieljcswalker@gmail.com (D.W.); melissa.a.jones@postgrad.curtin.edu.au (M.J.); susbinraj.wagle@postgrad.curtin.edu.au (S.R.W.); bozica.kovacevic@postgrad.curtin.edu.au (B.K.); j.chester@student.curtin.edu.au (J.C.); edan.johnston@student.curtin.edu.au (E.J.); 2 Hearing Therapeutics, Ear Science Institute Australia, Queen Elizabeth II Medical Centre, Perth, WA 6009, Australia; elaine.wong@earscience.org.au (E.W.); marcus.atlas@earscience.org.au (M.D.A.); 3 Department of Pharmacology, Toxicology and Clinical Pharmacology, Faculty of Medicine, University of Novi Sad, Hajduk Veljkova 3, 21101 Novi Sad, Serbia; mikovmomir@gmail.com

**Keywords:** bile acids, β-cells, microcapsules, diabetes mellitus, chenodeoxycholic acid, nano gel

## Abstract

Introduction: Recent studies in our laboratory have shown that some bile acids, such as chenodeoxycholic acid (CDCA), can exert cellular protective effects when encapsulated with viable β-cells via anti-inflammatory and anti-oxidative stress mechanisms. However, to explore their full potential, formulating such bile acids (that are intrinsically lipophilic) can be challenging, particularly if larger doses are required for optimal pharmacological effects. One promising approach is the development of nano gels. Accordingly, this study aimed to examine biological effects of various concentrations of CDCA using various solubilising nano gel systems on encapsulated β-cells. Methods: Using our established cellular encapsulation system, the Ionic Gelation Vibrational Jet Flow technology, a wide range of CDCA β-cell capsules were produced and examined for morphological, biological, and inflammatory profiles. Results and Conclusion: Capsules’ morphology and topographic characteristics remained similar, regardless of CDCA or nano gel concentrations. The best pharmacological, anti-inflammatory, and cellular respiration, metabolism, and energy production effects were observed at high CDCA and nano gel concentrations, suggesting dose-dependent cellular protective and positive effects of CDCA when incorporated with high loading nano gel.

## 1. Introduction

Diabetes mellitus is a condition that is characterised by chronic hyperglycaemia and inflammation, which is divisible into two primary classifications; type 1 diabetes (T1D) and type 2 diabetes (T2D) [1,2]. T1D is the result of autoimmune destruction of insulin producing β cells of the pancreas, whilst T2D is the result of decreased insulin sensitivity [2,3]. Diabetes is an extremely pervasive condition in Australia, affecting 4.8% of the population and placing significant economic burden on the health sector, accounting for 2.3% of all allocated health care expenditure in Australia [3,4].

Current treatments for diabetes predominantly rely on oral anti-hyperglycaemic agents or the subcutaneous administration of insulin to patients [1,5,6]. Insulin is used in almost all T1D patients and many T2D patients [2]. Insulin is useful in the regulation of blood glucose; however, it does not address the underlying aetiology of either T1D or T2D, nor the associated inflammation, oxidative stress, and cardio-metabolic sequelae [1]. Once commenced, patients will generally require insulin for the remainder of their life. Adherence and compliance of insulin administration can be an issue for many patients. In one study, 88.1% of patients were non-compliant in their insulin regimen [7]. Non-compliance then results in further complications. While replacing insulin alone is a useful treatment for diabetes, the ideal scenario will be replacing the redundant β cells with an alternative treatment that may provide more stable long-term glycaemic control for patients [8].

Microencapsulation is a technique that has been explored to deliver pharmaceuticals to patients, including the delivery of pancreatic islets [8,9,10,11,12,13,14,15,16]. There are several excipients that may be introduced into capsules to improve capsule properties, cell survival, and insulin release including bile acids (BA)s, sodium alginate (SA), and ultra-soluble gels (USG) [17,18,19,20,21].

BAs are steroid-like endogenous molecules that are synthesised in the liver and are used by the body to regulate glucose, lipids, and energy, as well as modulation of the immune system, nervous system homeostasis, and gut microbiota. BAs are also used to assist in the absorption of vitamins and lipids [21,22,23]. Diabetic mice have been found to be deficient in the primary BA chenodeoxycholic acid (CDCA), reinforcing BAs role in glucose control [24]. It is thought that, potentially, alteration of the BA pool may provide a novel method to control glycaemia in diabetic patients [25]. Additionally, BAs are highly desired excipients in drug delivery application due to their unique physico-chemical properties and stability [8,17,18,19,26,27,28,29,30,31,32,33,34,35]. Mooranian et al. were the first to reveal that the addition of CDCA to microcapsule formulations improved drug release kinetics of the anti-oxidant, anti-inflammatory, and anti-diabetic drug probucol (PB). CDCA was also able to improve durability of capsules within the gastrointestinal tract, catering for the creation of controlled, targeted drug delivery [9]. It is hypothesised that BAs have a stabilising effect on capsules by repelling water and by deprotonation of carboxylic acid within the capsule, this results in an increase electronegative ionic forces and high magnitude Zeta potential, and thus more stable delivery systems. Enhanced electrokinetic stability via Zeta potential modifications at the colloidal surface interface allows for improved structural stability via prevention of flocculation and Van der Waals mediated matrix stabilisation [8,9].

CDCA containing capsules have also been shown to improve encapsulated cell viability via calorimetric assays. CDCA based formulations were also shown to have fewer inflammatory markers, increased insulin secretion, and improved respiration compared to capsules without CDCA [8].

Sodium alginate (SA) is another excipient that aids in the delivery of drugs [36,37,38,39,40,41]. SA has been found to effectively coat capsules containing pancreatic β cells at a thickness that allows for the same insulin release profile as un-encapsulated cells [36]. Consistently, SA has been found to not stimulate the immune system, whilst allowing for appropriate glycaemic response in β cells in animal models [38,39,41,42]. Generally, SA is effective in the short-term protection of β cells but, eventually, inflammation and lack of engraftment lead to failure of the cells [37,41]. It is postulated that lack of adequate oxygen and nutrient supply, inefficient waste product exchange, macrophage-mediated surface decomposition, and fibrosis lead to the inadequate conditions for long term encapsulated cellular survival [37].

Ultra-soluble gels (USG) are non-petroleum based biological polymers that can be integrated into capsules to modify their physical properties [13,14,15,16,42,43,44]. They provide a suitably biocompatible microenvironment and are a relatively cheap way to vary properties of the capsules including their strength and consistency [42,43]. The type and proportion of the USG are the primary parameters used to vary these physical properties of the capsules [42,43].

A key issue with traditional encapsulation techniques is the lack of oxygenation and proper nutrient exchange for cells, which results in cellular hypoxia and apoptosis [45]. As well as the addition of BAs, alternative capsule formation methods have been investigated to improve the viability of these cells. Dispersed islet cell encapsulation is one such method that has shown potential [46]. Traditional methods involved the encapsulation of intact islets [45], while dispersed encapsulation attempts to suspend dispersed individual (or small groups) of cells [46]. Del Guerra et al. found that dispersed islet cell encapsulation had improved survival rates of cells, likely due to improved oxygenation [46]. Using the Buchi system and methodology established in our laboratory, capsules with improved morphology, surface topography and mechanical-osmotic stability were found at formation frequencies of >2000 Hz and <20 min bath gelation. This use of higher BA concentrations also contributed to increased cell survival [47].

This paper will explore the biological effects of increasing CDCA concentrations and the use of various solubilising nano gel systems and USGs on encapsulated β-cells.

## 2. Materials and Methods

### 2.1. Materials

Sodium alginate (Low viscosity SA, 99%), chenodeoxycholic acid (CDCA, 99%) and poly-l-ornithine hydrochloride (PLO; molecular weight 30–70 kDa) were purchased from Sigma chemical Co, (St. Louis, MO, USA). Calcium chloride dehydrate (CaCl_2_·2H_2_O, 98%) was obtained from Scharlab S.L, Australia. Dulbecco’s modified Eagle’s medium (DMEM) and other required supplements were purchased from Sigma Chemical Co (St. Louis, MO, USA). All other solvents and reagents were purchased from Merck (Bayswater, VIC, Australia) and were HPLC garde and used without further purification.

### 2.2. Microencapsulation Technology

Microencapsulation of cells was achieved with the Büchi Ionic Gelation Vibrational Jet Flow technology system with methods previously established in our laboratory [29,31,48,49,50,51,52,53,54,55,56,57,58]. Briefly, by using concentrations of 1.5 × 10^6^ cells/mL as the encapsulating medium, freshly resuspended cells were mixed with an encapsulating hydrogel-polymer system in a 1:1 ratio, whereby the mixture was then used to produce cell-loaded microcapsules via vibrational jet flow technology, coupled with frequency-modulated laminar jet flow [59]. The microcapsules were formed via ionic-gelation co-complexation using divalent cations of calcium chloride in an aqueous solution stirred at 100 RPM stabilised at 37 degrees Celsius.

Cells were encapsulated into eight different formulations. Each formulation contained 1.5% SA and 2% poly-l-ornithine hydrochloride (PLO), as well as various concentrations of USG in order to solubilise CDCA as necessary (part of method development, to ensure consistent solubility of CDCA was obtained). Stock solutions were mixed thoroughly prior to cell encapsulation. Control formulations (1, 3, 5, and 7) contained no CDCA while test formulations (2, 4, 6, and 8) contained various concentrations of CDCA. Specifically, all pair of formulations contained 0.2, 0.4, 0.8, and 1.6% of USG, respectively, while test formulations (2, 4, 6, and 8) contained 0.3, 0.6, 1.2, and 2.4% of CDCA, respectively. Encapsulation occurred in a bath of 2% CaCl_2_. Using Dulbecco’s modified eagle medium (Gibco Life Technologies, USA) NIT-1 [60] cells were grown in 25 cm^2^ flasks (Thermo Fisher Scientific, Melbourne, Victoria, Australia). The medium also contained 5% penicillin-streptomycin (Thermo Fisher Scientific, Melbourne, Victoria, Australia) and 10% foetal bovine serum [8,49,50].

### 2.3. Morphology and Topography Studies

Established quantities of newly encapsulated cells were mounted onto glass slides with a calibrated scale, observation of capsule morphology was observed via light microscopy with a 4× objective lens. Light microscopy was performed with an Olympus IX-51 inverted microscope (Olympus Company, Shinjuku, Tokyo, Japan) [8,29].

MIRA3 FibSEM (Tescan, Brno, Czech Republic) was used for scanning electron microscopy (SEM) surface assessment of the capsules with a 2.5 nm resolution at 3 kV. Capsules were coated in platinum at a thickness of 5 nm while under vacuum to observe capsule topography [8,29].

Cell viability studies within the capsules were also undertaken via microscopy. Cells were stained with CellTrace carboxyfluorescein succinimidyl ester (Life Technologies, Carlsbad, CA, USA) to determine the number of living cells. Living cells could then be counted with UltraVIEW Vox spinning disk confocal microscope (Perkin Elmer, Waltham, MA, USA) and Yokogawa CSU-X1 (Perkin Elmer, USA).

CDCA distribution was also performed with tetramethylrhodamine isothiocyanate, a compound that fluoresces when under UV light. Distribution was observed with the UltraVIEW Vox spinning disk confocal microscope (Perkin Elmer, USA) at 532 nm.

### 2.4. Sizing

Capsule sizes were determined with two methods; Mie and Fraunhofer scattering via the Mastersizer 2000 (Malvern Instruments, Malvern, UK) and spectroscopy using the Zetasizer 3000HSa (Malvern Instruments, Malvern, UK) using previously established methods [9].

### 2.5. Cell Viability Assays

In addition to viability studies via microscopy, they were also carried out prior to encapsulation. A 40 µL aliquot was removed from a cell mix after re-suspension and centrifugation. 10 µL of 0.4% tryptophan blue (Sigma Chemical CO, Melbourne, Victoria, Australia) was then added and mixed. A total of 10 µL was then added to each side of a Countess counter chamber slide (Invitrogen, Seoul, South Korea). A Countess Automated Cell Counter (Invitrogen, Seoul, South Korea) was then used to determine the number of viable cells [9].

### 2.6. Insulin Production and MTT

The MTT (3-[4,5-dimethylthiazol-2-yl]-2,5 diphenyl tetrazolium bromide) assay is another technique used to more efficiently screen cell viability by assessing metrics of mitochondrial activity [61]. A total of 5 mg/mL MTT stock was prepared and used within 24 h of being made. A 96 well plate (Thermo Fisher Scientific, Melbourne, Victoria, Australia) had 20 µL of stock MTT solution added to each well. Each well had newly made microcapsules added. After a 48-h incubation, insulin concentration was calculated with the use of mouse insulin ELISA (Mercodia Cooperation, Uppsala, Sweden). The data was then normalised based on the viable cell count within the capsules.

### 2.7. Inflammatory Markers

Capsules were cultured for 48 h before 100 µL aliquots were removed to investigate any inflammatory markers released by encapsulated cells using the BD Biosciences CBA technology (BD Biosciences, Franklin Lakes, NJ, USA). CBA analysis was used to detect tumour necrosis factor α (TNF-α), interferon γ (IFN-γ), interleukin-6 (IL-6) and interleukin-1β (IL-1β) with the use of Mouse BD Flex Sets (BD Biosciences, Franklin Lakes, NJ, USA) via Attune Acoustic Focusing Flow Cytometer (Life Technologies, Carlsbad, CA, USA). The data was then normalised based on the viable cell count within the capsules.

### 2.8. Mitochondrial Activity

The Seahorse Flux Analyser XF 96 (Seahorse Bioscience, North Billerica, MA, USA) was used to measure various mitochondrial parameters in real time assays using a method developed by the laboratory [8,49,50]. Parameters measured included: extracellular acidification rate (ECAR), oxygen consumption rate (OCR), proton production rate (PPR), ATP production (ATPP), Basal Respiration (BR), Coupling Efficiency (CE), Maximum Respiration (MR), Glycolysis (G), Non-Mitochondria-OCR (NM-OCR), non-glucose-derived extracellular acidification rate (NGD-ECAR), Spare Respiratory Capacity (SRC), and Proton Leak (PL). The data was then normalised based on the viable cell count within the capsules.

## 3. Results

### 3.1. Microscopy

**Figure 1:**
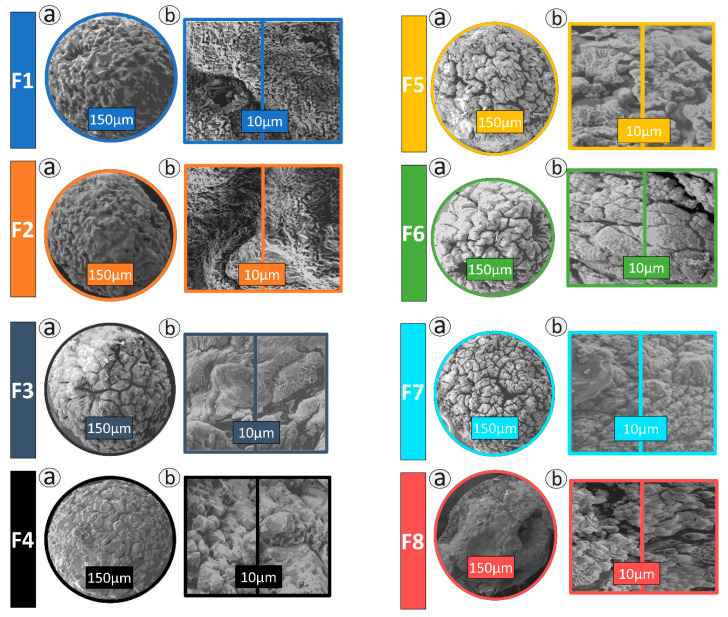
Topography and sizing of encapsulated cells by SEM for F1–F8 at magnifications of 150 μm and 10 μm.

Formulations F1–F8 show consistent ovoid/spherical morphology with comparable surface topography shown in Figure 1. Capsules all have consistent sizing overall at ~850 µm as measured by Mie and Fraunhofer scattering and spectroscopy. Surface topography of F8 showed slightly more ovoid and crenated morphology with fewer pores. This is consistent with our previous work, which showed CDCA produces consistent capsules with fewer pores [8,9]. CDCA also has been previously shown to form more dense capsules, which accounts for the slight difference in shape [9]. Crystalline structures within the surface topography are in line with work that showed they are Cl, O, and Ca atoms, which assist in the microcapsule formation [9,33,62]. Previous work using the same encapsulation procedure found uniform distribution of β-cells within the layers of the capsule [8,33].

### 3.2. Insulin Production and MTT

**Figure 2:**
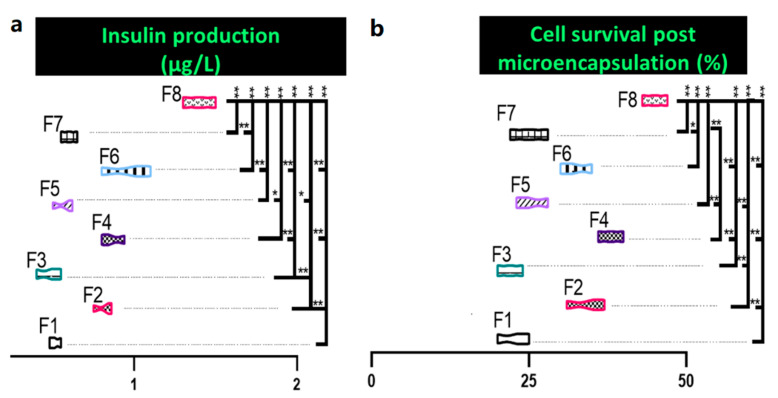
Insulin production (**a**) and cellular survival of encapsulated cells (**b**). Data are mean ± SEM, *n* = 3. * *p* < 0.05, ** *p* < 0.01.

Insulin production was recorded in μg/L and is presented in Figure 2a. Both insulin production and cell survival were highest in F8. In comparison to control formulation F1, insulin production was significantly increased by >200% post cell-count normalisation, as outlined in Figure 2a (*p* < 0.01). Formulations containing CDCA showed significantly increased insulin production when compared to their paired formulations without CDCA. Cell survival was determined by MTT assay (Figure 2b). Overall, survival rates were less than optimal, with no formulation having >50% survival. Formulations containing CDCA (F2, F4, F6, and F8) were all significantly higher than their non-CDCA counterparts, save F6, which was not significantly higher than F5.

### 3.3. Inflammatory Markers

**Figure 3:**
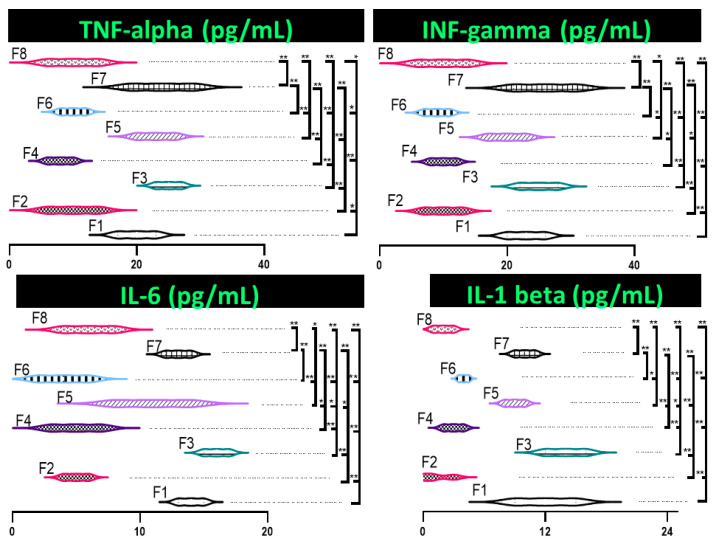
Inflammatory markers of encapsulated cells. Cytokines include tumour necrosis factor-alpha (TNF-alpha), intrerfeuron-gamma (IFN-gamma), interleukin-6 (IL-6), and interleukin-1 beta (IL-1 beta). Data are mean ± SEM, *n* = 3. * *p* < 0.05, ** *p* < 0.01.

IL-6, TNF-α, IFN-γ, and IL-1β were reduced in CDCA-containing formulations (Figure 3). TNF-α decreased by 50% in F8 compared to control (*p* < 0.05), IFN-γ and IL-6 decreased by >50% (*p* < 0.01), and IL-1β decreased by > 80% (*p* < 0.01). A trend appeared with this decrease in inflammatory markers is consistent with our previous work. It is also suggested to be a contributing factor to the increased cell viability in the CDCA containing capsules [8]. No significant variance of cytokine concentrations of TNF-α, IFN-γ, IL-6, or Il-1β emerged based on formulation concentrations of CDCA.

### 3.4. Mitochondrial Respiration, Metabolism, and Bioenergetics

**Figure 4:**
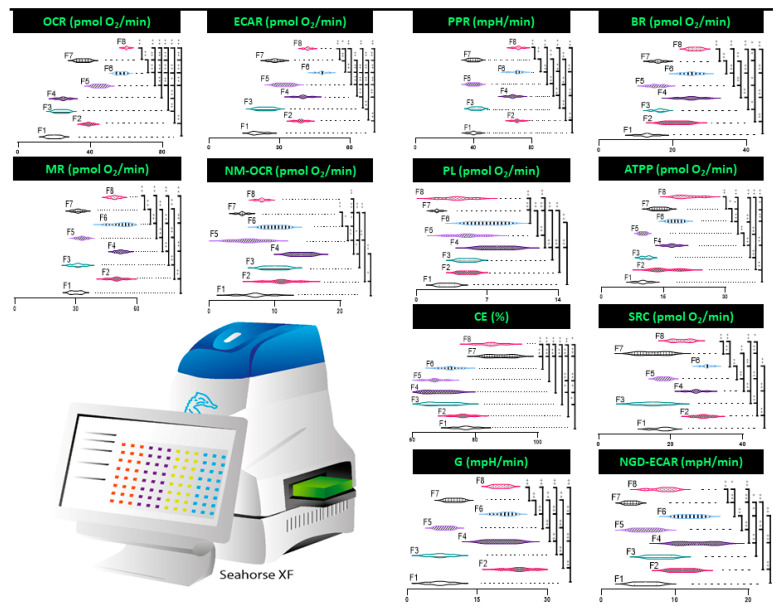
Cellular respiration parameters of encapsulated cells as measured by Seahorse XF platform. Measurements are oxygen consumption rate (OCR), extracellular acidification rate (ECAR), proton production rate (PPR), basal rate (BR), m rate (MR), non-mitochondrial oxygen consumption rate (NM-OCR), proton leak (PL), adenosine triphosphate production (ATPP), coupling efficiency (CE), spare respiratory capacity (SRC), glycoloysis (G), and non-glucose derived extracellular acidficication rate (NGD-ECAR). Data are mean ± SEM, *n* = 3. * *p* < 0.05, ** *p* < 0.01.

Encapsulated cells containing CDCA showed an increase in mitochondrial activity as measured by the Seahorse CF analyser (Figure 4). The results indicate in aerobic respiration that appears to correlate with the presence of CDCA within the capsule formulation. This increase is in mitochondrial energy use and respiration is supported by previous work performed in our laboratory with CDCA based capsules [8]. However, distinct oscillating inter-formulation differences are observed in many areas investigated, such as: oxygen consumption rate (OCR, pmol O_2_/mL), proton production (PPR mpH/min), basal rate (pmol O_2_/mL), and metabolic rate (pmol O_2_/mL). This variability raises the possibility that slight adjustment to microcapsule microenvironment through adjustment of bile acid levels influences cellular energetics in a multiplexed manner.

## 4. Discussion

### 4.1. Insulin Production and MTT

BAs, including CDCA, have previously shown the ability to improve the diffusion of drugs, including insulin, in capsules [63]. Due to the amphipathic nature of BAs, they are able to form micelles that improve the solubilization of insulin [64]. Insulin-BA conjugates are also thought to improve insulin solubility [65]. Increased solubility has likely resulted in increased insulin diffusion across the capsule, and thus increased insulin production measurements. Increased insulin production, correlating with increased CDCA, is supported by previous research performed in by our laboratory. We previously indicated that this may be a direct result of CDCA interacting with the β-cells [8]. In addition to being diffusion enhancers, BA such as CDCA also stimulate increased insulin production from β-cells. BAs are known to stimulate several intra and extracellular receptors. For example, Lee et al. found that BAs (including CDCA) are able to activate the Farnesoid X Receptor (FXR), which is expressed in pancreatic β-cells [66]. FXR stimulation primarily functions as a negative feedback receptor for BA synthesis [67]. FXR activation has also, however, been shown in animal models to reduce the signs of diabetes through increased insulin production from pancreatic β-cells and subsequent glucose regulation. Insulin production was stimulated through FXR activation through genomic and non-genomic mechanisms [68]. FXR has also been shown to reverse insulin resistance in animal models, suggesting it may be beneficial in the treatment of T2D [69]. Interestingly, increased levels of BAs in the plasma of mice was also found to decrease insulin sensitivity through an undetermined mechanism; however, exogenous administration of insulin was found to have no effect on insulin sensitivity [70]. It may be that CDCA is improving both insulin production and diffusion within the capsules.

This poor survival rate of encapsulated (and non-encapsulated) cells recorded is consistent with other work that suggests that poor survival is the result of inadequate nutrient and oxygen exchange within the cells [45]. CDCA containing capsules did, however, significantly improve the cell survival rates compared to cells without CDCA (*p* < 0.01). Increased survivability of cells within CDCA microcapsules may have occurred through activation of the FXR receptor. Activation of FXR by CDCA has previously stimulated anti-oxidant and detoxifying pathways [71]. CDCA is also thought to improve the transport of nutrients and oxygen into the cells to further sustain them [8]. Conversely, CDCA has been found to be toxic in primate models. This relationship was found to be largely dose related [72]. Additionally, some BAs have been shown to induce Ca^2+^ influx into cells which trigger cell apoptosis [73]. Previous studies by our laboratory have, however, also confirmed the protective effects and improved cell viability of CDCA [8]. It is also possible that a decrease in inflammatory markers (Figure 3) may contribute to increased cell viability. However, further studies are required to determine the protective mechanisms of CDCA in cells to assist in further improving cell viability.

### 4.2. Inflammatory Markers

Elevated levels of TNF-α have been associated with insulitis and associated white cell infiltration. While not directly linked to β cell apoptosis, TNF-α is thought to contribute to cell death [74]. Even low-level increases in proinflammatory cytokines such as TNF-α have been previously associated with reduced β cell function and decreased insulin production. This factor may be an underlying cause of the significant increase in insulin production seen in F8. FXR activation has been associated with a down regulation in genes that results in production of inflammatory markers such as TNF-α [75]. Consistent decrease in inflammatory cytokines in formulations may be caused by a potential anti-inflammatory effect of CDCA. CDCA has been shown to decrease the production of inflammatory cytokines in murine models. In particular, it was found to decrease production of IL-4 and IL-5, as well as proinflammatory cytokines such as TNF-α [76]. In the case of murine models of inflammatory bowel disease, TNF-α production was attenuated by the activation of the FXR receptor, in which CDCA is a potent agonist [77]. In vitro, both CDCA and DCA have shown anti-inflammatory capabilities via the inhibition of TNF-α synthesis in monocytes [78]. Conversely, CDCA previously given to patients with irritable bowel syndrome increased the presence of inflammatory markers in the terminal ileum, including IL-6 and TNF-α. The mechanism for this action was not entirely elucidated, but it was hypothesised that these effects were influenced by BA dosage [79].

While neither IFN-γ nor TNF-α alone can induce cell death, it has been found that, when combined, they induce apoptosis in islet cells by interfering with calcium channels [80]. IFN-γ expression has also been shown to be influenced by BAs, such as in the case of white blood cells. When administered to patients, ursodeoxycholic acid (UDCA) has demonstrated a suppressive effect on IFN-γ production, whilst CDCA administration has exerted a proliferative effect [81]. In addition to decreasing TNF-α concentrations in monocytes, FXR activation in lung tissue exerts a suppressive effect on TNF-α, IFN-γ, and proinflammatory gene expression [82]. FXR knockout mice possess increased inflammatory markers including IFN-γ, TNF-α, and IL-6 [82]. The relationship between FXR activation and reduction in biomarkers associated with inflammation is becoming more apparent with the growing body of research, which highlights the potential anti-inflammatory benefits of bile acids in pathologies associated with chronic inflammation, such as diabetes mellitus.

IL-1β, in combination with IFN-γ, has also been found to initiate apoptosis in β cells. When combined with IFN-γ, IL-1β was found to impair the endoplasmic reticulum, which led to cell death [83]. IL-6 has also been shown to induce apoptosis in β cells. At 20ng/mL, IL-6 was found to induce nitric oxide production which is commonly associated with apoptosis [84]. CDCA has also shown dose-dependent inhibition of IL-1 and IL-6 production in monocytes. At concentrations of 60μmol/L and 80μmol/L CDCA was able to block 50% of IL-1 and IL-6 production in cell culture, respectively. At 250 μmol/L, CDCA was completely inhibitory of IL-1, IL-6, and TNF-α [85]. Inhibition of IL-6 production by BAs has been found to occur through several mechanisms, including inhibition of STAT3 and blocking caspase-dependent IL-6 synthesis [86].

In summary, it is likely that the activation of FXR by CDCA and directly interfering with inflammatory marker synthesis resulted in decreased inflammatory markers. This decrease may also contribute to improved cell viability.

### 4.3. Mitochondrial Respiration, Metabolism, and Bioenergetics

Glycolytic pathways showed increased activity, with ECAR, PPR, and G all increasing in F8 compared to F1. PL remained consistent between F1 and F8. Glucose is broken down into lactate and H+, which increases ECAR and PPR. Increased G is, therefore, the primary contributing factor to increased ECAR and PPR; however, it is important to note that CO_2_ from aerobic respiration can also contribute to ECAR [87]. Given that NGD-ECAR did not show any significant change between F1 and F8, it suggests most of the total ECAR is the result of increased G. G increased by >100% in F8 compared to the control (*p* < 0.01). ECAR and PPR also increased as expected due to the increased G (*p* < 0.01). In line with the changes found in ECAR and PPR, CDCA treatment of pancreatic cells has previously been shown to decrease pH (and thus increase ECAR and PPR) [88]. PL relates to the proton that leave the mitochondria without producing ATP; increases in PL have been linked to cardiac disease [88]. As such, the consistent PL between F1 and F8 suggests that there is no increase in ATP production inefficiency. Decreased favouring of glycolysis has previously been found in cells treated with CDCA. In adipose tissue CDCA increased the rate of aerobic rather than glycolytic pathways for energy production. This increased oxidation was found in hyperglycaemic conditions, and is thought to contribute to a CDCA’s ability to decrease obesity. [89]. Our study, however, saw an increase in both G oxidative pathways for energy production.

Increase in oxidative pathways for energy production were also demonstrated by the seahorse analyser. OCR increased by ~200% in F8 compared to control, which indicates increased aerobic energy production (*p* < 0.01). NM-OCR remained stable; this suggests that the predominant source of the increased OCR was from the mitochondria. Given this, it follows that other oxidative parameters also increased. BR increased by 100% between F8 and the control, and MR also increased (*p* < 0.01). CE slightly increased, indicating improved oxidative efficiency (*p* < 0.05). SRC remained consistent. Evidence supporting the increased aerobic mitochondrial activity by BAs has previously been established. TGR5 is a G–protein coupled receptor that is also activated by BAs including CDCA [90]. Broeders et al. found that activation of TGR5 by CDCA in animal models was linked to increased mitochondrial activity, as measured by increased energy and oxygen depletion. Increased mitochondrial activity was also indicated by a 5% higher basal metabolic rate in CDCA treated animals [91]. Adipocytes treated with TGR5/FXR agonists have also been shown to significantly increase oxygen consumption, but it also decreased ATP production [92].

Overall energy production in the form of ATP doubled when comparing F1 and F8 (*p* < 0.01). Increased ATP production has been found in rat livers treated with BAs. UDCA was found to increase ATP production by 278% in the liver [93]. Increased ATPP is likely due to the increase in both glycolytic and oxidative pathways, as previously established.

In opposition to the findings of this study, CDCA has also been found in previous research to be cytotoxic to mitochondria. CDCA was found to increase mitochondrial membrane permeability and mitochondrial depolarization, and decrease mitochondrial hydrogenase activity. Such results, however, are largely dosage dependent [94]. In cardiac muscle, 52.5 nmol/mg CDCA was found to cause significant mitochondrial dysfunction [95]. This suggests further research may be required to determine the therapeutic and toxic doses for CDCA to minimise side effects in potential future patients.

Increased mitochondrial metabolic activity has also been linked to increased insulin production, which may further explain the increase in insulin production found in this study. In particular, increased Ca^2+^ production as a result of increased mitochondrial activity acts as a stimulus to increase the activity of glucose induced insulin production [96]. Increased mitochondrial activity is also associated with increased cell survival by decreasing reactive oxygen species formation and encouraging cell longevity [97].

In future, it is envisaged that the nanogels loaded with cells would be administered via the portal vein in suitable recipients, using protocols and guidelines as per the Edmonton Protocol [98]. Recipients may experience improved quality of life without significant immunosuppression-related complications.

To summarize, energy expenditure and mitochondrial respiration increased with USG and CDCA concentration. There are several possible mechanisms for this, including the activation of TGR5 by CDCA. This increased activity may contribute to the increased insulin production and cell survival rate.

Although the short-term biological effects of CDCA on the cells were positive, its long-term effects remain unknown. It is plausible to assume that, after acute application of CDCA and cell activation, the cells will return to normal biological functions and maintain healthy haemostasis. It is also possible that CDCA effects will last beyond its exposure, and may even have detrimental effects on the cells, in the long-term. All such effects, beyond the duration of the experiment remain to be studied.

## 5. Conclusions

To conclude, capsule morphology and topographic characteristics remained relatively consistent, irrespective of CDCA or USG concentrations. Improved insulin production, inflammatory, cellular respiration, and energy production parameters were seen at increased CDCA and USG concentrations in pancreatic β-cells. This study suggests a dose-dependent effect of CDCA when incorporated with high loading nano gel in microcapsules. Further studies are required to confirm this improved performance of CDCA and USG containing capsules in vitro.

## Data Availability

The data presented in this study are available on request from the corresponding author.
